# An interesting case of pacemaker endocarditis

**DOI:** 10.1007/s12471-019-01309-9

**Published:** 2019-07-25

**Authors:** K. K. Sahu, A. K. Mishra, A. A. Sherif, A. Doshi, B. Koirala

**Affiliations:** 1 Department of Internal Medicine, Saint Vincent Hospital, Worcester, Massachusetts United States; 2 Department of Cardiovascular diseases, Saint Vincent Hospital, Worcester, Massachusetts United States; 3 Department of Internal Medicine, Reliant Medical Group, Saint Vincent Hospital, Worcester, Massachusetts United States

An 81-year-old male with a past medical history significant for coronary artery disease with status post percutaneous coronary intervention, complete heart block with permanent pacemaker, presented for the complaints of generalised weakness, fever, shortness of breath and a cough with expectoration of one-month duration. He denied any chest pain, palpitation, urinary symptoms, pyuria or other signs of urinary tract infection. Patient also had an indwelling catheter for chronic urinary retention secondary to neurogenic bladder. His vitals showed tachypnoea (respiratory rate 24/min) and increased heart rate (pulse rate 90/min). His labs showed leucocytosis of 28,000 cells/mm^3^ with 11% band cells, lactic acidosis and evidence of acute kidney injury. Urine analysis showed leucocyte esterase positivity with sediment showing moderate bacteria and white blood cells. Chest X‑ray was negative for any consolidation or pulmonary congestion. Samples of blood, sputum and urine were sent for cultures and he was started on ceftriaxone and azithromycin. Fig. 1a shows sheep blood agar growing small bacterial colonies with clear alpha hemolysis. Within 24 hours of sampling, his sputum cultures showed growth for gram-positive cocci (GPC) in clusters, blood cultures showed growth for GPC in tetrads and clusters (Fig. 1b) and urine culture grew gram-negative rods.

**Fig. 1:**
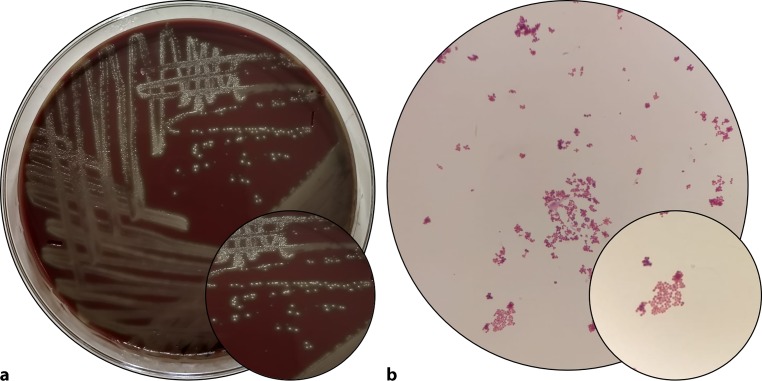
**a** 5% sheep blood agar, showing alpha haemolytic colonies, approximately 1–2 mm in size; **b** Microscopic image of gram staining of colonies showing gram-positive cocci of approximately 1 mm in size arranged in clusters (*arrow*)

What is the working diagnosis? Which microbes are responsible for bacteraemia and what is the likely source of infection?

## Answer

You will find the answer elsewhere in this issue.

